# The Effect of a SEL (Social–Emotional Learning) Intervention Program Based on Emotional Regulation and Metacognitive Awareness for Special Education Preservice Teachers Experiencing Adapted Teaching in Mathematics

**DOI:** 10.3390/ejihpe14070133

**Published:** 2024-07-09

**Authors:** Stella Gidalevich, Ella Mirkin

**Affiliations:** Department of Special Education, Oranim Academic College of Education, Kiryat Tivon 3600600, Israel; allamirkin@gmail.com

**Keywords:** emotional regulation, metacognitive awareness, SEL intervention program, preservice teachers, reappraisal, suppression

## Abstract

This study’s aim was to examine the influence of a unique intervention program based on emotional self-awareness and the development of emotional regulation as an important component of SEL combined with metacognitive awareness. Seventy-two third-year preservice teachers participated for one year, tutoring a mathematically challenged student. This intervention was applied to an experimental group where each participant learned to assess his/her emotional state using a special ruler prior to teaching a lesson, and according to the ruler’s assessment results, the preservice teacher was assisted by a variety of emotional regulations as well as metacognitive strategies. A control group learned only metacognitive strategies. This study’s results indicated that experimental group participants showed notable improvement in cognitive reappraisal post-intervention compared to the control group, which showed no change. For metacognitive awareness, in both research groups, an increase was apparent post-intervention. Nevertheless, a comparison of the effect size of differences between the two measurement points indicated that the experimental group exhibited a greater improvement in metacognitive regulation compared to the control group. A significant positive correlation was found in the experimental group between cognitive reappraisal and metacognitive regulation. Assimilation and training of emotional skills among preservice teachers during training are necessary.

## 1. Introduction

One of the biggest challenges among teaching educators and educational policymakers in training programs around the world is equipping future teachers for the complex reality in the field of education. According to research by the Central Bureau of Statistics [1], in 2020–2021, there was a dramatic increase of about 60% in the drop-out rate of teachers after two to five years on the job. Studies have shown that preservice teachers need support to develop psychological and emotional resilience, as well as specific strategies to maintain their health and efficiency in this demanding profession [2]. Trainers of preservice teachers and new teachers often feel overwhelmed in the field of education, for example, because of the complex variety of student needs and the lack of quality instructions [2].

One of the ways to prevent stress overload and burnout and reduce teacher dropout is to develop social and emotional skills (SEL) for teaching preservice teachers during their training years. These skills are critical because they equip future teachers with strategies of how to deal with students’ behavioral needs, develop relationships with students, and effectively manage classrooms [3,4]. In the professional literature, it was found that current and future teachers will behave more effectively if they learn to evaluate themselves regularly and develop their social and emotional skills [5]. Social–emotional learning is the process by which people—at any age—understand and manage their emotions, set positive goals and work to achieve them, feel and express empathy for others, develop healthy identities, establish and maintain positive relationships, and make decisions in a responsible and caring manner [6]. Jones and Bouffard [7] argued that SEL skills can be classified into three broad categories: social/interpersonal skills, cognitive regulation, and emotion-related skills. Teachers experience a wide range of significantly powerful emotions at work, interacting with students, parents, colleagues at school, and vis-à-vis educational policy [8].

Emotional competence is defined as a set of basic skills that include identifying, expressing, and regulating emotions of self and others [9]. Emotional competence plays an important role in well-being, work engagement, and teaching self-efficacy and can predict teacher retention, attrition, and perceptions of classroom management [10].

Most of the previous research on this topic focused on the ability of teachers to promote emotional competence in their students and not in themselves [11]. The general assumption is that by increasing emotional skills, it is possible to reduce teachers’ experiences of stress at work, thus preventing mental illnesses and preventing the high rate of attrition among teachers [12].

The current study is unique in that it aims to develop an intervention program to provide preservice teachers in their third year of training with tools for emotional regulation during teaching in the practicum and to examine the effectiveness of this intervention.

**Emotional Regulation as a Central Element in Social–Emotional Learning**

Emotional regulation is a central element in SEL awareness, understanding, and regulation of emotion within the school context and beyond. It contributes to teachers’ teaching effectiveness, the quality of the relationship between student and teacher, and general job satisfaction [13].

According to Gross [14], emotional regulation is a process that affects human emotions, referring to an internal experience and an external expression of these emotions. Gratz and Roemer [15] stated that teachers achieve emotional regulation based on three abilities:

(1) Awareness, understanding, and acceptance of emotions: Numerous studies have shown that expressing feelings and articulating emotional situations in words can relieve anxiety, anger, and stress [16].
(2) Control of impulsive behaviors and behavior according to desired goals: The well-known longitudinal study involving the marshmallow experiment showed that children who managed to delay gratification at the age of four developed into better students, people with richer social lives, and higher self-confidence than those who failed to resist the temptation [17].
(3) The use of appropriate emotional regulation strategies to meet personal goals.

Gross [18] indicated two main emotional regulation strategies that people use: reappraisal and suppression. Reappraisal is defined as a renewed cognitive evaluation, referring to a person’s ability to change a negative emotion into a positive one by changing their way of thinking about the situation with the help of a renewed interpretation, thereby reducing the negative impact of the emotion [14]. Repression is defined as a delay in external behavior to express emotion. Gross [18] argued that reappraisal can reduce physiological and behavioral responses. However, repression does not affect the experience of the emotion but increases a physiological effect. In a study conducted among 393 preservice teachers, it was found that the suppression strategy was mainly explained by low emotional awareness and difficulty describing emotions. Students with low emotional awareness of their feelings and with difficulty finding words and describing their feelings preferred to use the suppression strategy to regulate their feelings [19]. Catastrophizing, self-blame, or negative thinking are associated with a high level of anxiety and depression, while positive reappraisal and planning strategies are associated with a low level of anxiety and depression [20]. The findings of the study conducted among 2710 teachers in the United States showed that the repression strategy has a harmful effect on teachers’ well-being. The teachers who adopted a strategy of hiding their real feelings from their students are expected to adopt suppression as their usual way of regulating emotions. Recommendations from this study were to encourage teachers to learn to express real feelings and employ a strategy of re-evaluation of their feelings to respond positively to various situations in the classroom [21].

In the current study, we proposed an intervention that fosters preservice teachers’ awareness of emotions before teaching and a variety of strategies for emotional regulation by changing overwhelming emotions that make teaching difficult.

**Metacognitive Awareness**

Cognition is the process involved in knowing, which refers to the use of simple strategies like memorization and information processing and higher-level strategies such as problem-solving and critical thinking. Metacognition is a form of cognition, a second or higher-order thinking process for understanding the task and the solution strategy [22,23,24,25]. According to Schraw and Dennisson [26], metacognitive awareness enables a person to plan, sequence, and monitor his or her learning so that the improvements can be seen directly in their performance.

Metacognitive awareness contains two aspects: metacognitive knowledge and meta- cognitive regulation [26,27]. Metacognitive knowledge is composed of declarative knowledge about the strategy/task (“what”?), procedural knowledge used in various cognitive strategies (“how”?), and conditional knowledge (“when”? and “why”?) that is important for the flexible and adaptive use of various cognitive strategies [24,28].

Metacognitive regulation refers to a dynamic strategic processing activity that helps control one’s thinking or learning [29] through three phases. In the planning (forethought) phase, learners set goals for their planning of specific activities to complete a specific task. Next, in the monitoring (performance) phase, learners use their goals to monitor the process and move it along, using their goals as checkpoints for progress on tasks. Finally, in the evaluation phase, learners use the information gained from the completed task to improve their performance on the next task, including what worked, what did not work, and why [25]. Metacognitive knowledge belongs predominantly to domain-specific strategies, and metacognitive regulation is domain-general for processing strategic activation and application via the three phases [23,24]. 

Researchers in various fields have recognized that teachers with high metacognitive awareness have greater influence on the learning process of their students. Therefore, integrating practices for the development of metacognitive awareness [30,31] into teacher training programs is highly recommended. This practice was examined in a sequence of previous studies dealing with the training of mathematics teachers (e.g., [32]). Still, it has been shown that although they received training for developing metacognitive awareness among their students, these skills were not developed among the teachers themselves [33]. A potential explanation for this, found by previous research, shows that preservice teachers tend to overestimate their own metacognitive awareness compared to the level of metacognitive awareness estimated by their instructors or researchers [34].

Past studies dealing with SEL skills and with emotional competence and emotional regulation in particular have recommended the integration of dedicated courses in teacher training programs. At the same time, a similar recommendation was heard from theorists whose field of research is focused on metacognition.

There are a few studies that deal with the combination of these two fields (metacognition and emotional regulation) within one study. For example, the purpose of a recent study [35], in which 238 preservice teachers participated, was to examine the correlation between metacognition and emotional regulation. In this study, a distinct effect was found between metacognition and the ability to regulate emotions and, specifically, between metacognitive knowledge and reappraisal as a strategy for emotional regulation. The researcher’s recommendation is to apply reappraisal to deal with negative emotions because metacognition allows you to re-evaluate the negative experience [35]. These findings are supported by a study conducted among 1283 older Germans in their 40s [36]. The research findings indicate that a clear relationship was found between a metacognitive strategy of metacognitive temporal coping (among other things, focusing on a positive past or a good future or minimizing negative thoughts and feelings) and a strategy of re-evaluation in emotional regulation, but no relationship was found with a strategy of suppression.

To date, these two areas have not been studied in combination with the intervention program among preservice teachers. The **purpose of the current study** is to investigate the effect of a **holistic intervention** that is designed to provide a solution for both the development of emotional regulation skills and the cultivation of metacognitive skills on preservice teachers’ emotional regulation and metacognitive awareness. The intervention was implemented among third-year preservice teachers during a practical experience in adapted mathematics teaching classes taught individually by the research participants in inclusive schools.

**Research Questions and Hypotheses:**

1. Does a SEL-based intervention program affect preservice teachers’ emotional regulation ability when expressed at a higher level of reappraisal and less suppression of emotions?
2. Does a SEL-based intervention program affect preservice teachers’ metacognitive awareness when expressed at a higher level of metacognitive knowledge and metacognitive regulation?
3. Does preservice teachers’ improvement in emotional regulation correlate with their improvement in metacognitive awareness?

We hypothesize that our proposed intervention plan will elevate emotional regulation in the experimental group. This hypothesis stems from the conclusions of previous studies on improving SEL abilities in general and the ability to regulate emotions in particular, due to a direct intervention designed specifically for this purpose [4,12].

We will not hypothesize about the contribution of the intervention on metacognitive awareness due to the lack of sufficient empirical evidence regarding the relationship between emotional regulation and metacognitive awareness. Several studies have recently been conducted indicating the existence of a relationship between metacognition and emotional regulation [35,36]. However, it should be taken into account that preservice teachers tend to value their metacognitive awareness highly, and, thus, a ceiling effect of their high report may be created [34]. For these reasons, we will not hypothesize about the correlation between preservice teachers’ improvement in emotional regulation with the improvement in their metacognitive awareness.

## 2. Materials and Methods

**Participants**

The participants were recruited from an education training college located in a large district in northern Israel. The sample size was determined a priori by using v. 3.1.9.7 G*power software. To conduct an ANOVA with repeated measures (within/between interaction) analyses, using the test parameters (2 study groups, 2 time-point measures, low–medium effect size = 0.27, ηp^2^ = 0.07, α error = 0.01, and a medium correlation coefficient between the two time points = 0.4), the total sample size required was at least 66 participants. In order to decrease the risk for multiple comparison that may increase the likelihood of incorrectly rejecting a null hypothesis and making a type I error [37], the significance level was restricted to 0.01. We implemented the Bonferroni correction method and divided the α probability by 4 (the number of testing variables). Considering the sample size required, 72 special education preservice teachers in the 3rd year of their training for the B. Ed. degree were recruited to participate in this study (2 men and 70 women), aged between 20 and 27 (M = 23.49; SD = 1.81). 

The participants were divided randomly into two groups. The experimental group comprised 39 preservice teachers (1 man and 38 women) (see “Intervention Program Structure”). The control group comprised 33 preservice teachers (1 man and 32 women) (see “Intervention Program Structure”). In order to examine whether the two groups differed in gender, a _χ_2 test was conducted. No significant difference was found in gender in the two study groups (_χ_2(2) = 0.01, *p* = 0.905). The percentage of men and women who participated in this study did not differ significantly across the two study groups. In both groups, nearly the entirety of the sample were women. Finally, the *t*-test for two independent samples indicated no significant difference in age between preservice teachers who were assigned to the experimental group (M = 23.47; SD = 1.98) and preservice teachers who were assigned to the control group (M = 23.52; SD = 1.62) (t(70) = 0.09, *p* = 0.925).

**Intervention Program Structure and Fidelity of Implementation**

Each preservice teacher in the two research groups was asked to teach math to one student in a series of individually adapted classes for an entire academic year. This experience was a part of their clinical training for the B. Ed. program in special education at the teaching college. Notably, in the two years preceding this study, the preservice teachers experienced teaching in classes in inclusive and special schools.

The preservice teachers who participated in the current study were divided into four experimental groups of 18 students. Each group worked within one elementary school in the northern part of the country, where students taught math to grades 1 to 6. In each school, nine preservice teachers were randomly assigned to the experimental group and nine preservice teachers to the control group. This division was made to avoid a bias of the differences that exist between the four schools.

The four schools were chosen based on their nurture index = 6. A nurture index of 1 is indicative of a school populated by students from a high socioeconomic background, while a nurture index of 10 indicates a school in which most students are of low socioeconomic status. A school’s nurture index is determined based on 4 factors: parents’ education, parents’ income, geographical area, and country of birth.

Before the beginning of the school year, an appeal was made to the school principal and mathematics team, requesting that they select 18 children who had the greatest difficulty compared to their peer group. Each preservice teacher was assigned to one child for the entire year (on a weekly basis). In the first two sessions (two hours each), the preservice teacher conducted a curriculum assessment in mathematics (in relation to the curriculum) according to the pupil’s age and built a personal curriculum according to the assessment findings and the curriculum in the pupil’s class.

The preservice teacher then tutored the pupil for two hours once a week until the end of the year. At the end of the school year, a summative assessment was made. It should be noted that building a personal study plan, aimed on the one hand at reducing private studies and, on the other hand, integrating the pupil with what is learned in his class, is a challenge for all preservice teachers.

On behalf of the college, each group was accompanied by a pedagogical instructor who has a Ph.D. in education and is an expert in the field of adapted teaching in mathematics. The age range of the instructors ranged from 37 to 41 (M = 44.50; SD = 5.80), and their seniority ranged from 15 to 26 years (M = 20.75, SD = 4.57). Each instructor accompanied 18 preservice teachers in one school, 9 from an experimental group and 9 from a control group, to control for a bias in instruction style.

The duties of the instructor were as follows:

1. Assisting in the construction of personal study plans for all preservice teachers.
2. Conducting observations in the preservice teacher’s lessons every week.
3. Holding a half-hour personal meeting for training once a fortnight with each preservice teacher to reflect on the previous lesson, help focus the goals of the lesson, and choose teaching strategies for the following lessons. Frequent and professional accompaniment of this type has been found to be effective in previous studies (e.g., [38]).
4. Inspection of the lesson plans and teaching materials for all preservice teachers.

At the same time, at the college, the preservice teachers studied an annual course, “Tools and Strategies for adapted teaching for Students with difficulties in mathematics in elementary school”. This course was taught in three groups by three lecturers with a Ph.D. in education and field expertise with work experience in the field of education in adapted teaching in mathematics. In this course, teaching strategies were taught, metacognitive skills of the preservice teachers were developed, and they were guided to develop metacognitive skills among the students.

The preservice teachers were exposed to SEL programs throughout each year of training: in the first year, the foundations of the program are reflected in their action research. In the second year of their training, preservice teachers participating in the study attended an annual course in the field of self-regulated learning, where they learned about the importance of developing self-regulation skills among students, received tools for self-regulation, and prepared and implemented an individual/group intervention program involving a SEL principal for students in special education schools where they had experience. In the 3rd year, they were required to be involved in the emotional work with students who have learning difficulties. However, the preservice teachers were not instructed in their own self-regulation and did not learn tools for their self-emotional regulation.

In light of the fact that the preservice teachers for two years acquired tools to work in the SEL field with the students, it was decided to check if the SEL intervention program with the preservice teachers themselves would lead to changes; that is, was it enough to train the preservice teachers to develop SEL skills among students and would they know how to transfer the tools and skills to themselves?

The **experimental group** (half of each group in all four schools): the SEL intervention program extended a total of 16 personal meetings throughout one school year, once every two weeks for 10 min. Each preservice teacher met with a pedagogical instructor for a 10-minute training SEL intervention. In light of the literature review and recommendations from previous studies (for example, [6]), the goal of the intervention program was to embody two key components of emotional competence among preservice teachers: awareness of emotions and their impact and the acquisition of tools promoting choice for response. The program began after the completion of the evaluation phases and the construction of personal curricula by the preservice teachers for their students.

**Figure 1:**
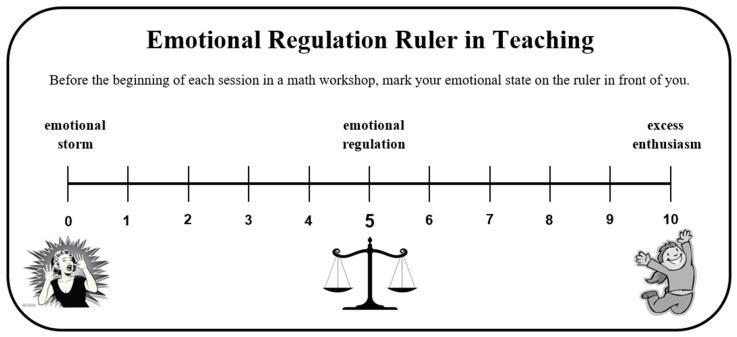
Emotional regulation ruler in teaching.

A pedagogical instructor accompanying the group met personally with each preservice teacher in the experimental group and gave them a ruler for emotional regulation (see Figure 1).

The choice of a ruler was made following the studies in which this tool was found to be effective among students [39,40]. In the current study, an adjustment was made for preservice teachers in the design of the idea.

**Figure 2:**
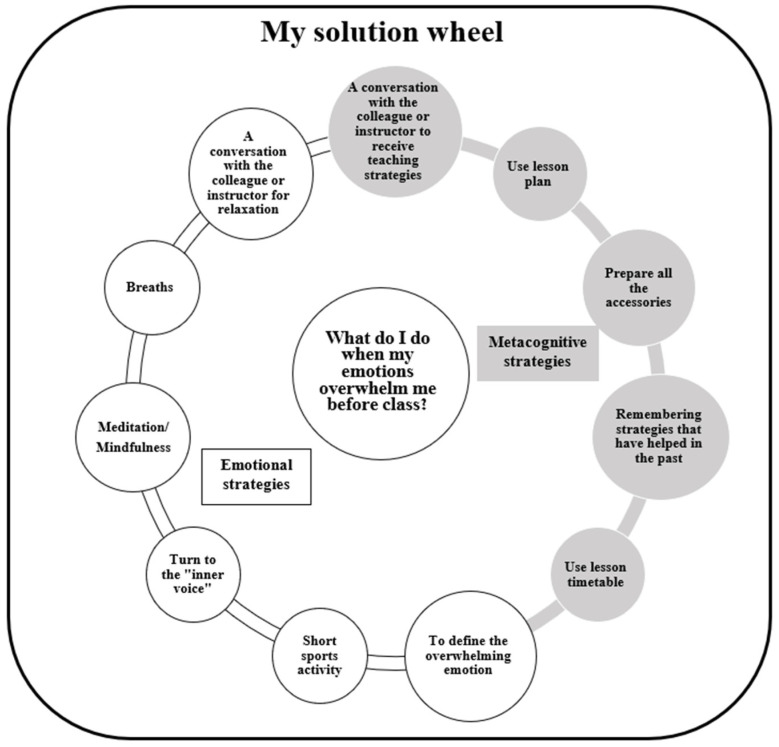
The solution wheel that the preservice teacher could use for emotional regulation.

In this personal meeting, each preservice teacher was instructed to use the ruler: before each lesson with the student, the preservice teacher must assess her own emotional state. If before the class the preservice teacher feels emotionally regulated, she will be able to start teaching. If she evaluates herself dealing with a storm of emotions or with over-enthusiasm, she was offered a wheel of solutions for emotional regulation, which was also shared with every preservice teacher in the experimental group (see Figure 2).

The solutions in the wheel were divided into **metacognitive strategies** and **emotional strategies** that the preservice teacher could use for emotional regulation. After using the strategies, if the preservice teacher managed to regulate herself, she was instructed to start teaching. During the first month, preservice teachers received reminders to follow these directives from their instructors via WhatsApp before each lesson. In the personal conversations with the instructors throughout the school year, the preservice teachers in the experimental group were also asked to reflect on the use of the emotional regulation ruler and the solution wheel.

Some solutions in the wheel refer to relaxation, breathing, and turning to the inner voice, following the recommendations of previous studies on the integration of the principles of self-efficacy on emotional well-being and mental and physical health [30,41], and some refer to integrating these principles among preservice teachers [11]. In addition, a solution that refers to sports activity is anchored in the research literature and indicates that physical activity leads to the creation of new brain cells to reduce cell damage [42]. 

In order to exhaust the potential of the solution wheel and find maximum solutions, the researchers met with three pedagogical instructors (10 to 15 years of experience in pedagogical guidance) in the field of adapted teaching, three researchers in the field of emotional regulation who published articles in this field, three practicing teachers of adapted teaching, and three fourth-year preservice teachers who had this experience in the year previous to research. Moreover, in personal conversations with the preservice teachers, aiming to explain the wheel, each preservice teacher was asked if she had any solutions to add and answered in the negative.

In this group, training was preservice teacher-centered, aiming to:

1. Cultivate emotional self-awareness;
2. Develop the preservice teacher’s emotional regulation strategies and affect self-image and self-control abilities;
3. Develop the preservice teacher’s metacognitive teaching strategies.

In the **control group**, which also contained half of each group at the school, the preservice teachers received the same number of personal sessions with the instructor and were exposed to the same metacognitive teaching strategies as in the experimental group. The difference between the groups is reflected in the fact that these preservice teachers were not exposed to the ruler for emotional regulation and strategies for emotional regulation. In other words, in this group, the intervention plan was preservice teacher-centered and focused on the development of the preservice teacher’s metacognitive teaching strategies.

**Figure 3:**
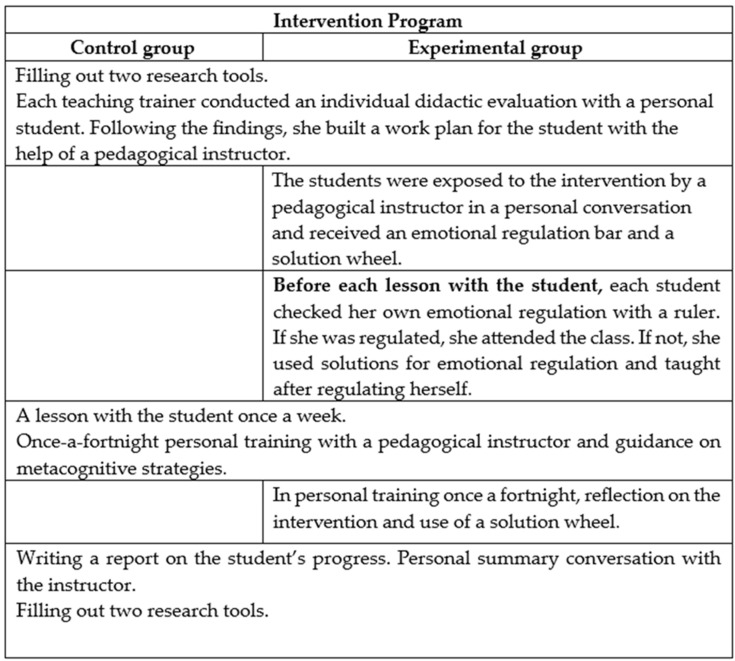
Summary of the research design.

Figure 3 summarizes the study design and the differences in the intervention between the two study groups.

**Materials and Instruments**

The preservice teachers answered emotional regulation and metacognitive awareness questionnaires at two time points (T1 and T2).

**Emotional Regulation.** The Emotion Regulation Questionnaire (ERQ) developed by Gross and John [43] consists of two subcomponents that measure the habitual use of reappraisal or suppression using a 7-point scale, from 1 (“strongly disagree”) to 7 (“strongly agree”). The reappraisal subcomponent consists of 6 items (e.g., “When I want to feel more positive emotions (such as joy or amusement), I think of something else”; “I control my emotions by changing the way I think about the situation I’m in”). The suppression subcomponent consists of 4 items (e.g., “I keep my emotions to myself”; “When I am feeling negative emotions, I make sure not to express them”). Since the items in the questionnaire were not validated to date among Israeli preservice teachers, we examined the construct validity of the items in this questionnaire using an EFA. 

We used an EFA using Varimax rotation to construct orthogonal factors based on an eigenvalue greater than 1. The results indicated that the 100 items of the ERQ questionnaire were constructed of three orthogonal factors that accounted for 70.90% of the variance. The items and factor loadings for the ERQ measure are presented in Table 1.

As can be seen in Table 1, the second and third factors of the ERQ questionnaire contained only two items each. These results contradict the recommendation by Little et al. [44] to retain only factors with at least three items. Therefore, an additional EFA analysis limited to two orthogonal factors using Varimax rotation was conducted on the ERQ questionnaire items. The results of the additional EFA are presented in Table 2.

As can be seen in Table 2, the results of the EFA analysis indicated that the two orthogonal factors of the 10 ERQ items explained a total variance of 58.53%, with each factor explaining at least 23% of the additional variance. In addition, the results of the EFA indicated that there were at least three items in each factor. This result takes into consideration the recommendation by Little et al. [44] to retain only factors with at least three items. The 10 items were divided into two factors, in accordance with the original subcomponents presented by Gross and John [43]. The reappraisal subcomponent consists of 6 items, and the suppression subcomponent consists of 4 items. 

Finally, the internal consistency of the two factors was examined. Cronbach’s alpha for the cognitive reappraisal subcomponent was α = 0.85, and for the suppression subcomponent, it was α = 0.74.

**Metacognitive Awareness in Preservice Teachers**. The self-report Metacognitive Awareness Inventory for Teachers (MAIT) was developed by Balcikanli [45]. This questionnaire contained 24 items, which produced two principal factors [27]: metacognitive knowledge and metacognitive regulation. The preservice teachers were requested to answer the 24 items on a five-point Likert scale ranging from 1 (“strongly disagree”) to 5 (“strongly agree”). Cronbach’s alpha reliability for the 24 items was α = 0.91, α = 0.83 for metacognitive knowledge, and α = 0.87 for metacognitive regulation.

**Research Ethics**

This study began after receiving approval from the ethics committee of the researchers’ academic institution. The purpose of this study was explained to the preservice teachers in order to obtain their written consent. Any preservice teacher was entitled to stop participating in this study at any time.

## 3. Results

Prior to examining the research questions and hypotheses, we examined whether the two preservice teacher groups differed significantly in their emotional regulation and metacognitive awareness at T1. A one-way MANOVA for the two emotional regulation subcomponents and for the two metacognitive awareness subcomponents indicated no significant differences between the two preservice teachers groups at T1 (F(2,69) = 1.92, *p* = 0.155, ηp^2^ = 0.05 and F(2,69) = 1.95, *p* = 0.150, ηp^2^ = 0.05, respectively). Table 3 presents the mean, SD, and F-values for each emotional regulation and each metacognitive awareness subcomponent at T1.

Therefore, in order to examine the difference between the preservice teachers’ emotional regulation and metacognitive awareness, two-way (2 × 2) mixed ANOVAs were conducted for each subcomponent. The independent variables were group (as the between-subjects factor) and time (as the within-subjects factor). The dependent variables were the emotional regulation and metacognitive awareness subcomponents. We hypothesized that the interaction between group and time would be significant for all subcomponents, meaning that the difference in the preservice teachers’ emotional regulation and metacognitive awareness between the two time points would differ according to group assignment. In order to examine the interaction source, we examined the simple effects by investigating the differences between the two time points in each preservice teachers’ group and calculated the Cohen’s d effect size of these differences.

### 3.1. Differences in Preservice Teachers’ Emotional Regulation and Metacognitive Awareness 

#### 3.1.1. Cognitive Reappraisal and Suppression

**Figure 4:**
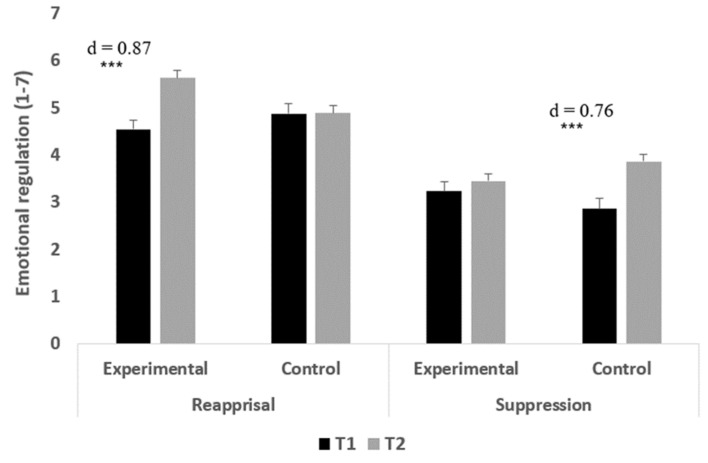
Means and standard errors (SEs) of the preservice teachers’ emotional regulation at the two-time points. *** *p* < 0.001.

- Cognitive reappraisal: The main effect of time was significant, F(1,70) = 12.95, *p* < 0.001, and ηp^2^ = 0.16, indicating higher cognitive reappraisal at T2 (M = 5.29; SD = 1.03) compared to T1 (M = 4.69; SD = 1.22). The main effect of group was not significant, F(1,70) = 1.01, *p* = 0.317, and ηp^2^ = 0.01. Finally, in accordance with the first hypothesis, the interaction of group and time was significant, F(1,70) = 12.25, *p* < 0.001, and ηp^2^ = 0.15. Examining the differences between the two time points in each preservice teachers’ group indicated that while the preservice teachers who were assigned to the experimental group exhibited significantly higher cognitive reappraisal at T2 compared to T1, no significant difference in cognitive reappraisal level was found among the preservice teachers in the control group (t(38) = 5.42, *p* < 0.001, Cohen’s d = 0.87 and t(32) = 0.06, *p* = 0.949, Cohen’s d = 0.01, respectively).
- Suppression: The main effect of time was significant, F(1,70) = 14.19, *p* < 0.001, and ηp^2^ = 0.17, indicating higher suppression at T2 (M = 3.64; SD = 0.87) compared to T1 (M = 3.06; SD = 1.20). The main effect of group was not significant, F(1,70) = 0.02, *p* = 0.897, and ηp^2^ = 0.00. Finally, in accordance with the first hypothesis, the interaction of group and time was significant, F(1,70) = 5.85, *p* = 0.018, and ηp^2^ = 0.08. Examining the differences between the two time points in each preservice teachers’ group indicated that while the preservice teachers who were assigned to the control group exhibited significantly higher suppression at T2 compared to T1, no significant difference in suppression level was found among the preservice teachers in the experimental group (t(32) = 4.38, *p* < 0.001, Cohen’s d = 0.76 and t(38) = 0.96, *p* = 0.341, Cohen’s d = 0.15, respectively). Figure 4 presents the means and standard errors (SEs) of the preservice teachers’ emotional regulation at the two time points.

As can be seen in Figure 4, the effect size of the difference between the two time points in the cognitive reappraisal among the experimental group is considered large, and the effect size of the difference between the two time points in suppression among the control group is considered medium/large [46].

#### 3.1.2. Differences in Preservice Teachers’ Metacognitive Awareness

- Metacognitive knowledge: The main effect of time was significant, F(1,70) = 22.29, *p* < 0.001, and ηp^2^ = 0.24, indicating higher metacognitive awareness at T2 (M = 4.22; SD = 0.45) compared to T1 (M = 3.92; SD = 0.50). The main effect of group was not significant, F(1,70) = 2.00, *p* = 0.162, and ηp^2^ = 0.03. Finally, as opposed to the second hypothesis, the interaction of group and time was not significant, F(1,70) = 1.56, *p* = 0.216, and ηp^2^ = 0.02.

The results indicated that both preservice teacher groups exhibited higher metacognitive knowledge at T2 compared to T1 (experimental: t(38) = 4.65, *p* < 0.001, and Cohen’s d = 0.75; control t(32) = 2.23, *p* = 0.033, and Cohen’s d = 0.39).

- Metacognitive regulation: The main effect of time was significant, F(1,70) = 39.49, *p* < 0.001, and ηp^2^ = 0.36, indicating higher metacognitive regulation at T2 (M = 4.46; SD = 0.42) compared to T1 (M = 4.00; SD = 0.58). The main effect of group was not significant, F(1,70) = 1.33, *p* = 0.252, and ηp^2^ = 0.02. Finally, in accordance with the second hypothesis, the interaction of group and time was significant, F(1,70) = 6.94, *p* = 0.010, and ηp^2^ = 0.09.

Examining the differences between the two time points in each preservice teachers’ group indicated that both preservice teacher groups exhibited significantly higher metacognitive regulation at T2 compared to T1 (experimental: t(38) = 7.23, *p* < 0.001, and Cohen’s d = 1.16; control: t(32) = 2.26, *p* = 0.031, and Cohen’s d = 0.39). However, a comparison of the effect size of the differences between the two time points indicated that the preservice teachers who were assigned to the experimental group exhibited greater improvement in metacognitive regulation compared to the control group.

**Figure 5:**
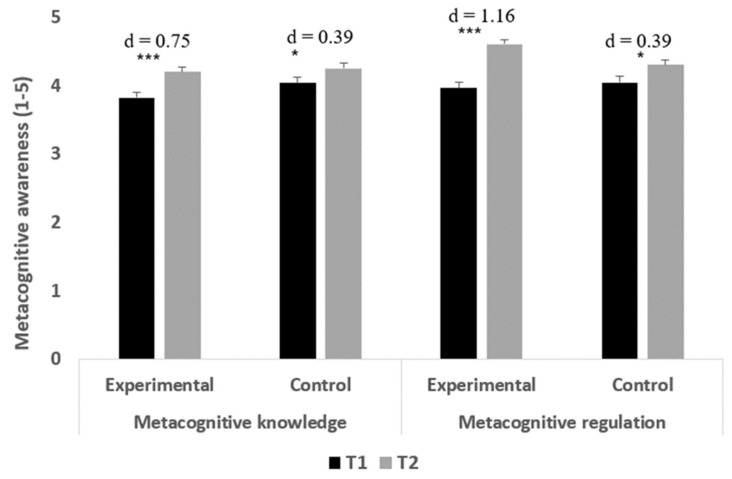
Means and standard errors (SEs) of the preservice teachers’ metacognitive awareness at the two time points. *** *p* < 0.001 * *p* < 0.05.

Figure 5 presents the means and standard errors (SEs) of the preservice teachers’ metacognitive awareness at the two time points. As can be seen in Figure 5, the effect size of the difference between the two time points in the metacognitive knowledge and the metacognitive regulation subcomponents among the experimental group were considered medium/large and large, respectively, and the effect size of the difference between the two time points in both metacognitive awareness subcomponents among the control group was considered small [46].

#### 3.1.3. The Correlation between the Improvement Rate of Emotional Regulation and Metacognitive Awareness

The current study examines the contribution of the SEL-based intervention program among preservice teachers regarding their emotional regulation ability and their metacognitive awareness. In order to examine whether the improvement rates in the subcomponents of emotional regulation and the subcomponents of metacognitive awareness correlate significantly, Pearson correlation analyses were conducted for each preservice teachers’ group separately. 

A significant positive correlation was found among the preservice teachers who were assigned to the experimental group between the improvement rate in the emotional regulation subcomponent—cognitive reappraisal—and the improvement rate in the metacognitive awareness subcomponent—metacognitive regulation (r(37) = 0.66, *p* < 0.001). The improvement rates in the subcomponents of emotional regulation and the subcomponents of metacognitive awareness did not significantly correlate among the preservice teachers who were assigned to the control group. The Fisher r-to-z transformation assessing the significance of the difference between two correlation coefficients indicated that the correlation coefficients between the improvement rate in the emotional regulation subcomponent—cognitive reappraisal—and the improvement rate in the metacognitive awareness subcomponent—metacognitive regulation—differed significantly between the two preservice teachers’ groups, Z = 2.73, *p* = 0.006. Table 4 presents the Pearson correlation coefficients on the improvement rate in emotional regulation and the improvement rate in metacognitive awareness in both groups.

**To summarize**, the results of the current study indicate that the preservice teachers who were assigned to the experimental group demonstrated higher cognitive reappraisal and higher metacognitive awareness levels. In comparison, the preservice teachers who were assigned to the control group also demonstrated a higher level of metacognitive awareness at T2, although they also demonstrated a higher level of suppression. In accordance with our first and second hypotheses, the improvement rates in the emotional regulation subcomponent—cognitive reappraisal—and in the metacognitive awareness—metacognitive regulation—subcomponent were significantly higher among preservice teachers from the experimental group compared to the control group (T2). Furthermore, the improvement rate in the emotional regulation subcomponent—cognitive reappraisal—and the improvement rate in the metacognitive awareness subcomponent—metacognitive regulation—significantly correlated only among preservice teachers from the experimental group. Thus, the more preservice teachers demonstrated higher improvement in their cognitive reappraisal level, the higher their metacognitive regulation level was, and vice versa.

## 4. Discussion

**Emotional regulation**. The intervention program in our study was based on a variety of strategies for emotional regulation connected with cognitive reappraisal that allowed flexibility in their use and adaptation to the situation, according to the recommendations of previous studies [21,47,48]. This program is designed to develop an awareness of cognitive reappraisal strategies. Knowing that these strategies are related to better mental health, people who use them show fewer signs of depression, are more satisfied and optimistic, have higher self-esteem, and have good interpersonal communication skills [43,49,50] among teachers as well [51,52]. The findings indicate that the program achieved its goals in the experimental group, where the participants demonstrated a significant improvement in cognitive reappraisal after the intervention, compared to the participants in the control group, where there was no change.

Another finding in our study indicates that in the **suppression** component, the participants in the control group showed a significant increase at the end of the intervention, while in the experimental group, there was no substantial change. In other words, the participants in the control group who did not learn to regulate their emotions with the help of cognitive reappraisal chose the path of suppression. This finding fits the findings of previous studies, according to which the suppression strategy is central among teachers who use it to regulate negative emotions that arise in their work [53].

Based on these findings, we may conclude that this unique intervention program constructed in our study aids future teachers in developing helpful emotional control strategies that use cognitive reappraisal instead of turning to the suppression strategies that are more common among teachers, which lead to anxiety and depression [20].

**Metacognitive Awareness**. Our findings show that, among both study groups, an increase was observed in the metacognitive awareness components after the intervention. The explanation for this lies in the structure of the intervention in the two research groups, in which all the participants received the metacognitive strategies. According to the recommendation of previous studies, during their training, the metacognitive development of preservice teachers should be supported, exposing them to metacognitive strategies several times through the course materials and training them to be more familiar with the strategies [33,54]. 

At the same time, the findings indicate that a comparison of the effect size of the differences between the two time points indicates that the preservice teachers in the experimental group exhibited greater improvement in metacognitive regulation compared to the control group. 

These findings can be explained by the fact that the ‘wheel of solutions’ used during the intervention in the experimental group allowed the preservice teachers to plan and regulate the teaching process after they evaluated themselves with the ruler. When they did not find themselves regulated, they planned and evaluated the emotional or metacognitive strategy they would use, and only after they felt regulated did they begin to teach. The actions of planning and control belong to metacognitive regulation [28], and thus the preservice teachers in the experimental group improved this component of metacognition, even if they chose an emotional strategy. Metacognitive skills help preservice teachers internalize and change the current emotional experience by anticipating a future outcome [55]. Metacognitive regulation helps preservice teachers in reappraisal while dealing with negative emotions [56]. 

Another finding indicates that a significant positive **correlation** was found among the preservice teachers in the experimental group **between** cognitive **reappraisal** and **metacognitive regulation**. The improvement rates did not significantly correlate among the preservice teachers in the control group. These results are consistent with previous research findings among the general population [36] and among preservice teachers [35].

## 5. Study Limitations and Future Directions

This study has several limitations. First, the number of participants in this study is relatively small and only represents special education preservice teachers. In addition, the sample includes mostly women (this reflects the gender representation in the teaching college where this study was conducted). Despite these limitations, the results of this study reinforce the concept that it is possible to develop awareness among preservice teachers of the emotions they feel before teaching and a variety of emotional regulation strategies to alter overwhelming emotions that make teaching difficult [4,12].

In future studies, it would be interesting to test an intervention in the field of emotional regulation in whole-class teaching rather than individual teaching. In addition, it would be interesting to check the preference of the specific strategies that the students used: mindfulness, breathing, talking with a friend, and more. Also, it would be interesting to conduct a longitudinal study that would test the strategies for emotional regulation one year after the end of the intervention.

## 6. Implications for Practice

In the current study, a unique intervention program was built that combines emotional regulation with metacognitive awareness, during which the preservice teachers were trained in emotional regulation strategies. Assimilation and training of emotional skills among preservice teachers during training are necessary, in addition to the integration of theoretical courses in the field [4]. In accordance with the research findings, we recommend that teacher training programs emphasize the development of emotional awareness and emotional regulation among preservice teachers and implement a variety of strategies for emotional regulation, such as defining an overwhelming emotion [16], mindfulness, breathing [41], and sports activity [42]. Such intervention programs would help preservice teachers not only in their exposure to a variety of strategies for emotional regulation but also in choosing effective strategies connected with cognitive reappraisal versus suppression strategies.

## Figures and Tables

**Table 1:** The results of an EFA analysis based on an eigenvalue greater than 1 for the ERQ items.

	Factor Loadings		
*ERQ Items*	1	2	3
I control my emotions by changing the way I think about the situation I’m in (Item 8).	0.86		
When I want to feel more positive emotion (such as joy or amusement), I change what I’m thinking about (Item 1).	0.82		
When I want to feel more positive emotion, I change the way I’m thinking about the situation (Item 7).	0.80		
When I want to feel less negative emotion, I think of something else (Item 10).	0.79		
When I want to feel fewer negative emotions (such as sadness or anger), I think of something else (Item 3).	0.62		
When I’m faced with a stressful situation, I make myself think about it in a way that helps me stay calm (Item 5).	0.56		
I control my emotions by not expressing them (Item 6).		0.88	
When I am feeling negative emotions, I make sure not to express them (Item 9).		0.81	
When I am feeling positive emotions, I am careful not to express them (Item 4).			0.93
I keep my emotions to myself (Item 2).			0.85
***Eigenvalue***	3.78	2.07	1.24
***Explained variance (%)***	34.29%	52.95%	70.90%

**Table 2:** The results of an EFA analysis restricted to 2 orthogonal factors for the ERQ items.

	Factor Loadings	
*ERQ Items*	1	2
When I want to feel more positive emotions, I change the way I’m thinking about the situation (Item 7).	0.84	
I control my emotions by changing the way I think about the situation I’m in (Item 8).	0.84	
When I want to feel fewer negative emotions, I change the way I’m thinking about the situation (Item 10).	0.80	
When I want to feel more positive emotions (such as joy or amusement), I think of something else (Item 1).	0.80	
When I’m faced with a stressful situation, I make myself think about it in a way that helps me stay calm (Item 5).	0.65	
When I want to feel fewer negative emotions (such as sadness or anger), I think of something else (Item 3).	0.60	
I keep my emotions to myself (Item 2).		0.83
When I am feeling positive emotions, I am careful not to express them (Item 4).		0.76
I control my emotions by not expressing them (Item 6).		0.71
When I am feeling negative emotions, I make sure not to express them (Item 9).		0.67
***Eigenvalue***	3.78	2.07
***Explained variance (%)***	35.30%	58.53%

**Table 3:** Mean, SD, and F-values for each emotional regulation and each metacognitive awareness subcomponent at T1 according to the preservice teachers’ group.

	Experimental Group		Control Group		F-Values		
*Study Measures*	M	SD	M	SD	F	P	η_p_^2^
**Emotional regulation subcomponents**							
Reappraisal	4.54	1.14	4.87	1.30	1.28	0.261	0.02
Suppression	3.23	1.11	2.86	1.27	1.70	0.196	0.02
**Metacognitive awareness subcomponents**							
Metacognitive knowledge	3.82	0.49	4.03	0.49	3.22	0.077	0.04
Metacognitive regulation	3.96	0.64	4.04	0.64	0.34	0.562	0.01

**Table 4:** Pearson correlation coefficients on the improvement rate in each emotional regulation subcomponent and the improvement rate in each metacognitive awareness subcomponent in both study groups.

	*Metacognitive Awareness Subcomponents*			
*Emotional Regulation* *Subcomponents*	Metacognitive Knowledge		Metacognitive Knowledge	
	Experimental	Control	Experimental	Control
Reappraisal	0.28	−0.07	0.66 ***	0.12
Suppression	0.05	0.17	0.30	−0.18

*** *p* < 0.001.

## Data Availability

The full datasets in this study are not available.
